# A cross-sectional study of the relationship between job demand-control, effort-reward imbalance and cardiovascular heart disease risk factors

**DOI:** 10.1186/1471-2458-12-1102

**Published:** 2012-12-21

**Authors:** Mia Söderberg, Annika Rosengren, Jenny Hillström, Lauren Lissner, Kjell Torén

**Affiliations:** 1 Department of Occupational and Environmental Medicine, Institution of Medicine, Sahlgrenska Academy, University of Gothenburg, Box 414, 405 30, Gothenburg, Sweden; 2 Department of Public Health and Community Medicine, Sahlgrenska Academy, University of Gothenburg, Box 414, 405 30, Gothenburg, Sweden; 3 Institute of Medicine, Sahlgrenska Academy, University of Gothenburg, SU/Östra, 416 85, Gothenburg, Sweden

**Keywords:** Psychosocial work environment, Cardiovascular heart disease risk factors, Job demand-control, Effort-reward imbalance, Gender

## Abstract

**Background:**

This cross-sectional study explored relationships between psychosocial work environment, captured by job demand-control (JDC) and effort-reward imbalance (ERI), and seven cardiovascular heart disease (CHD) risk factors in a general population.

**Method:**

The sampled consists of randomly-selected men and women from Gothenburg, Sweden and the city’s surrounding metropolitan areas. Associations between psychosocial variables and biomarkers were analysed with multiple linear regression adjusted for age, smoking, education and occupational status.

**Results:**

The study included 638 men and 668 women aged 24–71. Analysis between JDC and CHD risk factors illustrated that, for men, JDC was associated with impaired scores in several biomarkers, especially among those in high strain jobs. For women, there were no relationships between JDC and biomarkers. In the analysis of links between ERI and CHD risk factors, most associations tested null. The only findings were raised triglycerides and BMI among men in the fourth quartile of the ERI-ratio distribution, and lowered LDL-cholesterol for women. An complementary ERI analysis, combining high/low effort and reward into categories, illustrated lowered triglycerides and elevated HDL-cholesterol values among women reporting high efforts and high rewards, compared to women experiencing low effort and high reward.

**Conclusions:**

There were some associations between psychosocial stressors and CHD risk factors. The cross-sectional design did not allow conclusions about causality but some results indicated gender differences regarding sensitivity to work stressors and also how the models might capture different psychosocial dimensions.

## Background

Over the last decades there has been an accumulating amount of studies that have illustrated relationships between psychosocial work environment and health. One of the most influential models in this context is the Job Demand-Control (JDC) model [1,2]. Throughout the literature the combination high demands-low control, referred to as high strain, has frequently been linked to impaired health outcomes, e.g. cardiovascular and coronary heart disease [2-5], and psychological distress [6-8]. Another key model when examining work stress is the Effort-Reward Imbalance (ERI) at work structure [9]. ERI focuses on the reciprocity between efforts spent and rewards received. Similar to the JDC, exposure to this sort of work environment has been linked to negative health outcomes such as cardiovascular and coronary heart disease [10-13] and psychological ill-health [11,14].

However, while the ERI model leads to similar findings across studied populations [6,11,14,15], relationships between JDC exposure and health differ between examined groups. Throughout the JDC literature, most JDC to ill-health associations are found in blue-collar work environments dominated by men [1,3,5,6,8,12]. One plausible explanation is that the development of the JDC structure was mainly conducted among male blue-collar jobs, and is consequently adjusted to said job characteristics. When broadening study populations, more conflicting findings emerge. For example, little support has been found for female high-strain related ill-health [15,16]. Women exposed to high demand-high control (labelled as Active work) are more frequently related to health risks e.g. increased sick leave [17] and coronary heart disease risk [18] than those in high strain work. Furthermore, studies of both men and women in white-collar jobs have shown that; low demand-low control (passive jobs) could be associated with myocardial infarction [19] and increased inactive leisure time [20]. These heterogeneous findings emphasize the importance of examining general populations in order to capture general trends, rather than those that are group-specific.

Many studies evaluate relationships between psychosocial work environment and CHD [3,4,10,16], but few focus on how these stressors might relate to intermediate risk factors such as blood pressure, blood lipids or obesity [10,21-23]. This is important because these factors are related to lifestyle (diet, physical activity) and thus amenable to intervention. If work factors contribute, the scope for intervention by lifestyle modification might be influenced. Both JDC and ERI exposure have previously been linked to different CHD risk factors [12,24], especially high strain to increased blood pressure [25,26]. But although there are studies indicating associations, results are not consistent. In a Swedish cross-sectional study performed among a general population, all relationships between job demand/control and systolic blood pressure tested null [21]. The study also failed to illustrate links between psychosocial exposure and total cholesterol or BMI. This concurs with findings from another Swedish study by Alfredsson and colleagues [23] where no relationship of high strain to total cholesterol could be found. The study did, on the other hand, show relationships between ERI exposure and increased blood pressure and cholesterol (total cholesterol and LDL-cholesterol) [10]. Another study performed on the Whitehall II population reported links between the ERI stressors and ambulatory blood pressure in men [27]. Despite contradictive results, variables such as blood pressure and blood lipids are well-known biological risk-factors for CHD. If studying how these biomarkers interplay with psychosocial job environment, information on disease development might be found.

Further, the handful of studies in this context either examine specific populations or just use one psychosocial model [10,21-23]. Even though the JDC and ERI models partially overlap, they have complementary dimensions which could broaden the psychosocial scope. The JDC model mainly focuses on task characteristics, e.g. if work has to be performed fast or if volume of work can be mastered. The ERI variables also include measures of work task intensity, but since they originate from social exchange and organizational injustice theories [9], ERI also includes social aspects of work. The reward variable captures micro-social rewards, for example esteem and appreciation from colleagues and management. The reward factor also measures job security and promotion possibilities, which reflects macro-social perspectives, such as economic recession and company downsizing [9]. Hence including both models will provide a more diverse measure of psychosocial stressors.

Accordingly, this descriptive cross-sectional study aims to explore relationships between two complementary psychosocial structures and seven CHD risk factors in a general population. Men and women will be analysed separately given earlier results that indicate gender differences in health outcomes.

## Methods

### Procedure and participants

The collection of data was carried out from April 2001 until December 2003 and was a part of the INTERGENE and the ADONIX research projects. The sample selection was made by randomized sampling among the population of Gothenburg and surrounding metropolitan area, including all men and women aged 24–75. Selected subjects were mailed participant information, two questionnaires and an invitation to a basic clinical examination. Detailed information about the ADONIX and INTERGENE studies has been published [28,29]. Both studies were approved by the regional ethical review board of Gothenburg.

In all, 2492 subjects participated in the study. Out of those, 1991 participants completed the psychosocial questionnaire. Since analyses in this study were based on psychosocial work variables, and thus limited to subjects currently working, all participants on full-time sick leave, retired or unemployed were excluded. After these inclusion criteria 1306 participants remained. 49% of the subjects were men. Age ranged 24–71 with a mean age of 46.2 years (SD = 10.5).

### Measures

#### Cardiovascular heart disease risk factors

The CHD risk factors used in this study were diastolic blood pressure (DBP), systolic blood pressure (SBP), triglycerides, total cholesterol, HDL-cholesterol, LDL-cholesterol and body mass index (BMI). Measurements for all risk factors were gathered during the basic clinical examination which was conducted at a hospital in Gothenburg. All subjects were instructed to fast for 4 hours before attending. Body weight was measured to the nearest 0.1 kg and body height to the nearest cm with the subjects in light clothing and without shoes. Blood pressure measurements were carried out in a sitting position and after a 5-minute rest using an inflationary oscillometric blood pressure apparatus (Omron 711 Automatic IS). The blood pressure was measured two times and then the mean between the two was used. Blood samples were collected into tubes containing 0.1% EDTA for immediate serum lipids (total cholesterol, HDL-cholesterol, triglycerides) and plasma glucose analysis. Serum total cholesterol (TC) and triglyceride concentrations were determined by using enzymatic assays. LDL-cholesterol levels were estimated for all subjects with triglyceride levels under 4.00 mmol/L using the Friedewald equation.

#### Questionnaires

Analysed variables were captured with three different questionnaires. The first questionnaire’s main focus was allergy/asthma symptoms, but it also contained general information such as level of education and employment history items. The second questionnaire measured health and lifestyle variables, such as smoking, diet and medical history. The third questionnaire, which contained the majority of used variables in this study, was concentrated towards psychosocial work environment and psychological well-being.

#### Demand-control variables

The job demand-control items were based on a remodelled version [30] of Karasek & Theorell’s JDC structure. The authors behind the altered version label their measurements; work stress. The variables in their remodelled structure does, however, capture similar dimensions as job demand and job control and for that reason it will be referred to as job demand-control in this study. Both demand and control were explored with three items each. All items were scored using a scale (1–5) ranging from “Never” to “Almost all the time”. A sample demand item was, “How often during the last year has there been an increased amount of work?” An example of a control item was, “Do you have the possibility to decide over work tasks”. The demand and control variables were then added up separately. The sum of the score variables ranged between 3 and 15 for both variables. The median scores for both demand and control were 11. Each sum was dichotomized into high and low, using the median of the distribution as cut-off. The dichotomized variables were then combined into four categories and labelled, according to Karasek [1]; *high strain* (high demand-low control), *active* (high demand-high control), *passive* (low demand-low control) and *lowstrain* (low demand-high control) Often, when analysing JDC, only high strain is investigated in relationship to health outcomes. But since some studies show associations between other JDC type of exposure and impaired health outcomes, active and passive jobs will also be included in the analysis. Additionally, since this method is based on using summed up variable scores; participants with missing items were excluded, which resulted in 1227 remaining subjects.

#### Effort-reward imbalance

Effort-Reward Imbalance was measured with the Effort-Reward Imbalance at Work Questionnaire [3]. It is standard when using this instrument to exclude the item “My work is physically demanding” if the sample predominantly consists of white-collar workers. Since participants were employed in a broad variety of jobs, characteristics of occupational properties were investigated. Occupation among the sample was captured with one item “What is your occupation?”. The reply was then classified according to the International Classification of Occupations (ISCO-88) (ILO, 1990) [31] under the supervision of a senior occupational hygienist. The ISCO system makes it possible to organize jobs into sets of groups that could be categorized as either white or blue-collar jobs. It was then clear that the sample consisted of a majority of white-collar workers. Consequently, the mentioned item measuring physical labour was excluded. The sum of effort scores ranged from 5–25, with a median of 12. The reward items were all inverted and summed. Reward scores ranged from 17 to 55, with a median of 49. According to common praxis when analysing ERI, the two summed variables were divided (Σ_effort_/Σ_reward_) and then multiplied with a correction factor (0.4545), thus creating a ratio. A larger ratio indicates a greater imbalance between effort and reward. The ratio was then divided into categories, which were defined by the quartiles of the score distribution.

A complementary method for evaluating ERI, based on that of Siegrist and colleagues [11] was also used in this study. In this alternative analysis, the effort and reward variables were dichotomized by the median into high/low and then combined into four categories. Since there are no standard names for these combinations they were labelled as follows; ERI-1 (high effort and low reward), ERI-2 (high effort and high reward), ERI-3 (low effort and low reward), ERI-4 (low effort and high reward). Similar to the Siegrist and colleagues study [12] low effort-high reward (ERI-4) was regarded as the reference variable. The reason for using this altered method was based on an assumption that equal ratios may not relate to similar job experience e.g. low effort-low rewards can create a similar ratio as high effort-high reward. By using both methods it is possible to compare this study to other ERI-research, and also bring forth an additional perspective. Since sum scores were used for all ERI analysis, subjects with missing ERI items were excluded. The exclusion yielded a total of 1056 participants.

### Statistical analysis

Statistical calculations were performed with statistical software SAS (version 9.2 for Windows; SAS Institute; Cary; NC). When analysing JDC the four categories; high strain, active, passive and low strain were created. For the multiple regression analysis these categories were made into dummy variables, using low strain as a reference. When analysing ERI, categorical variables defined by the quartiles of the ratio-distribution was created and also turned into dummy variables. Investigation of the imbalance between effort and reward was made by comparing the first quartile (Q1) with the fourth (Q4). The reason for using the highest and lowest quartiles was to enhance imbalance differences. For the alternative ERI model; ERI-1, ERI-2, ERI-3 were used as dummy variables with ERI-4, capturing low effort and high reward, as reference variable. In all multiple linear regression between psychosocial models and CHD risk factors, men and women were analysed separately. All tests were double-sided with p-value <0.05 considered significant.

Each model was also adjusted for age, smoking, education and occupational status as potential confounders. Age was entered as a continuous variable. All other confounders were analysed as dummy variables given their categorical properties. The smoking variables comprised of; current smoker and ex-smoker with “never smoker” as reference. Education was categorized into; elementary school, lower secondary school, upper secondary school and university/higher education. Occupational status was based on the ISCO-88 [28] classification. According to this system subjects were classified into high skilled/low skilled white/blue-collar worker. The reference variable for education was “university education” and for occupation “high skilled white-collar workers” was used.

Analysis for JDC and ERI were both based on subjects with complete filled-in set of items for each model and consequently calculations constituted of different amount of subjects. For that reason, exploratory JDC analyses were made with the subjects used for ERI analysis, and ERI analyses with the participants from the JDC group. These analyses were made in order to eliminate possibilities that differences in results were due to different samples.

## Results

Characteristics of the sample according to JDC variables and gender are illustrated in Table 1 and characteristics according to ERI variables are illustrated in Table 2. 

**Table 1:** Characteristics of the sample for men and women according to job demand- job control variables

**Job demand-control**	**All**	**High strain**	**Active**	**Passive**	**Low strain**
		**High demand**	**High demand**	**Low demand**	**Low demand**
		**Low control**	**High control**	**Low control**	**High control**
**N**					
Men and women (n)	1227	246	198	402	381
Men	602	87	120	175	220
Women	625	159	82	227	161
**Mean Age** (**years**)					
Men	46.3	44.2	47.5	45.7	47.3
Women	45.4	46.6	44.6	45.3	44.5
**Current smokers**, **n** (%)*					
Men	84	15 (17.2)	19 (15.8)	26 (14.9)	24 (10.9)
v	135	36 (21.2)	12 (15.4)	54 (23.8)	33 (20.5)
**University education**, **n** (%) *					
Men	233	21 (24.1)	45 (37.5)	63 (36.0)	104 (47.3)
Women	289	82 (51.6)	46 (59.0)	82 (36.1)	79 (49.0)
**White**-**collar workers**, **n** (%)*					
Men	406	44 (50.6)	92 (76.7)	100 (57.1)	170 (77.3)
Women	551	149 (93.7)	69 (88.5)	195 (85.9)	138 (85.7)
**Blue**-**collar workers**, **n** (%)*					
Men	180	40 (43.0)	24 (20.0)	78 (39.6)	46 (20.9)
Women	50	7 (4.4)	6 (7.7)	25 (11.0)	12 (7.5)
**DBP** (**mmHg**) (**SD**)					
Men	81.1 (9.9)	82.2 (11.3)	82.0 (9.5)	81.5 (9.8)	80.0 (9.6)
Women	79.3 (10.1)	79.8 (9.0)	78.9 (10.2)	79.3 (10.7)	79.1 (10.4)
**SBP** (**mmHg**) (**SD**)					
Men	127.3 (16.1)	128.4 (18.5)	128.5 (16.0)	127.4 (16.3)	126.1 (14.8)
Women	119.0 (18.3)	118.2 (18.5)	120.1 (17.9)	119.3 (18.5)	119.0 (18.1)
**Triglycerides** (**SD**)					
Men	1.4 (0.9)	1.6 (0.9)	1.6 (1.1)	1.4 (0.9)	1.4 (0.7)
Women	1.1 (0.7)	1.1 (0.8)	1.0 (0.6)	1.1 (0.6)	1.1 (0.6)
**Total cholesterol** (**mmol**/**L**) (**SD**)					
	5.5 (1.01)	5.4 (1.0)	5.6 (1.0)	5.6 (1.0)	5.4 (1.0)
	5.3 (1.1)	5.3 (1.1)	5.3 (1.2)	5.3 (1.1)	5.3 (1.0)
**HDL**-**cholesterol** (**mmol**/**L**) (**SD**)					
Men	1.5 (0.4)	1.4 (0.3)	1.5 (0.4)	1.5 (0.4)	1.5 (0.3)
Women	1.8 (0.5)	1.8 (0.50)	1.9 (0.6)	1.8 (0.4)	1.8 (0.4)
**LDL**-**cholesterol** (**mmol**/**L**) (**SD**)					
Men	3.4 (0.9)	3.3 (0.9)	3.5 (0.8)	3.5 (0.9)	3.3 (0.9)
Women	3.0 (0.9)	3.0 (0.9)	2.9 (0.9)	3.0 (0.9)	3.0 (0.9)
**BMI** (**mmol**/**L**) (**SD**)					
Men	26.2 (3.4)	26.9 (3.3)	26.9 (3.5)	25.7 (3.2)	26.1 (3.5)
Women	24.6 (3.9)	24.6 (4.0)	24.7 (3.9)	24.5 (3.9)	24.7 (3.7)

* Column percentage.

**Table 2:** Characteristics of the sample for men and women according to effort-reward imbalance variables

**Effort-reward imbalance**	**All**	**ERI-1**	**ERI-2**	**ERI-3**	**ERI-4**
		**High effort**	**High effort**	**Low effort**	**Low effort**
		**Low reward**	**High reward**	**Low reward**	**High reward**
**N**					
Men and women	1056	315	212	222	307
Men	518	130	135	94	159
Women	538	196	77	128	148
**Mean Age** (**years**)					
Men	46.3	44.3	45.9	47.0	47.8
Women	45.6	45.6	44.4	45.0	46.6
**Current smokers**, **n** (%)					
Men	70	20 (15.4)	17 (12.6)	14 (14.9)	19 (12.0)
Women	112	37 (20.0)	17 (22.1)	35 (27.3)	23 (15.5)
**University Education**, **n** (%) *					
Men	202	53 (40.8)	58 (41.1)	41 (43.6)	54 (34.0)
Women	253	92 (49.7)	51 (66.2)	48 (37.5)	62 (41.9)
**White**-**collar workers**, **n** (%)*					
Men	348	85 (65.4)	103 (76.3)	54 (57.5)	106 (66.7)
Women	477	166 (89.7)	75 (97.4)	110 (85.9)	126 (85.1)
**Blue**-**collar workers**, **n** (%)*					
Men	156	39 (30.0)	28 (20.7)	38 (40.4)	51 (32.1)
Women	44	13 (7.0)	2 (2.6)	15 (11.7)	14 (9.5)
**Mean DBP** (**mmHg**) (**SD**)					
Men	81.1 (10.1)	80.1 (10.3)	80.9 (8.9)	81.2 (10.7)	82.0 (10.5)
Women	79.7 (10.2)	79.5 (9.9)	78.8 (8.8)	78.5 (11.0)	81.6 (10.5)
**Mean SBP** (**mmHg**) (**SD**)					
Men	127 (16.1)	125 (15.6)	127.2 (14.7)	126.7 (17.7)	128 (16.7)
Women	119 (18.4)	119.3 (19.4)	115.8 (15.9)	116.6 (16.7)	124.0 (18.9)
**Mean Triglycerides** (**SD**)					
Men	1.4 (0.9)	1.1 (0.8)	1.6 (1.2)	1.4 (1.0)	1.3 (0.7)
Women	1.1 (0.7)	1.17 (0.82)	0.9 (0.6)	1.0 (0.5)	1.1 (0.7)
**Total cholesterol** (**mmol**/**L**) (**SD**)					
Men	5.5 (1.0)	5.3 (1.1)	5.4 (1.1)	5.6 (1.0)	5.5 (1.0)
Women	5.4 (1.1)	5.31 (1.09)	5.4 (1.1)	5.4 (1.1)	5.4 (1.1)
**HDL**-**cholesterol** (**mmol**/**L**) (**SD**)					
Men	1.5 (0.4)	1.8 (0.3)	1.4 (0.3)	1.5 (0.4)	1.5 (0.4)
Women	1.8 (0.5)	1.7 (0.4)	2.0 (0.6)	1.8 (0.4)	1.8 (0.4)
**LDL**-**cholesterol** (**mmol**/**L**) (**SD**)					
Men	3.4 (0.9)	3.3 (0.9)	3.3 (0.9)	3.5 (0.9)	3.4 (0.9)
Women	3.1 (0.9)	3.0 (0.9)	2.9 (0.9)	3.1 (1.0)	3.1 (0.9)
**BMI** (**kg**/**m2**) (**SD**)					
Men	26.1 (3.2)	26.5 (3.4)	26.2 (3.3)	25.9 (3.4)	25.7 (2.9)
Women	24.8 (3.9)	25.0 (3.8)	24.4 (4.5)	25.0 (4.0)	24.5 (3.7)

* Column percentage.

Table 3 shows the results of the multiple linear regression between the job demand-control variables (high strain, active and passive) and CHD risk factors. Men exposed to a high strain work environment had higher scores for DBP (p = 0.02), SBP (p = 0.03) and triglycerides (p = 0.05). Men in passive jobs had significantly increased scores of total cholesterol and LDL-cholesterol. Additionally, total cholesterol was elevated among men in active jobs. In contrast to men, there were no significant relationships between any JDC variables and CHD risk factors among female participants. 

**Table 3:** Multiple linear regression for JDC and CHD risk factors

	** High strain**		** Active**		** Passive**	
	**Estimate(95%CI)**	**P**-**value**	**Estimate(95%CI)**	**P**-**value**	**Estimate(95%CI)**	**P-value**
DBP (mmHg)						
Men	3.3 (0.5 ; 6.0)	**0**.**02**	1.4 (−1.0 ; 3.8)	0.26	2.0 (−0.09 ; 4.2)	0.06
Women	0.5 (−1.9 ; 3.0)	0.68	−0.1 (−2.8 ; 3.0)	0.95	0.003 (−2.2 ; 2.2)	0.9977
SBP (mmHg)						
Men	4.6 (0.5 ; 8.8)	**0**.**03**	2.1 (−1.7 ; 5.8)	0.28	2.0 (−1.3 ; 5.3)	0.20
Women	−1.7 (−5.7 ; 2.3)	0.40	0.9 (−3.8 ; 5.7)	0.70	−1.3 (−5.0 ; 2.3)	0.47
Triglycerides (mmol/L)						
Men	0.2 (−0.002 ; 0.4)	**0**.**05**	0.06 (−0.1 ; 0.2)	0.45	−0.06 (−0.2 ; 0.1)	0.39
Women	−0.03 (−0.2 ; 0.1)	0.59	−0.002 (−0.2 ;0.1)	0.97	−0.01 (−0.1 ; 0.1)	0.74
Total Cholesterol (mmol/L)						
Men	0.1 (−0.1 ; 0.4)	0.31	0.2 (0.003 ; 0.5)	**0**.**04**	0.3 (0.06 ; 0.5)	**0**.**01**
Women	−0.2 (−0.4 ; 0.1)	0.21	−0.05 (−0.3 ; 0.2)	0.70	−0.1 (−0.4 ; 0.1)	0.20
HDL-cholesterol (mmol/L)						
Men	−0.04 (−0.1 ; 0.06)	0.45	0.02 (−0.08 ; 0.1)	0.74	0.04 (−0.04 ; 0.1)	0.36
Women	−0.03 (−0.1 ; 0.07)	0.55	0.04 (−0.1 ; 0.2)	0.52	0.02 (−0.1 ; 0.1)	0.73
LDL-cholesterol (mmol/L)						
Men	0.1 (−0.1 ; 0.4)	0.41	0.2 (−0.01 ; 0.4)	0.06	0.3 (0.1 ; 0.5)	**0**.**006**
Women	−0.1 (−0.3 ; 0.1)	0.35	−0.1 (−0.4 ; 0.1)	0.46	−0.1 (−0.3 ; 0.05)	0.15
BMI (kg/m2)						
Men	0.5 (−0.4 ; 1.4)	0.25	0.6 (−0.2 ; 1.4)	0.13	−0.4 (−1.1 ; 0.3)	0.22
Women	−0.2 (−1.1 ; 0.7)	0.64	−0.1 (−1.2 ; 1.0)	0.85	−0.7 (−1.5 ; 0.1)	0.10

High strain, active and passive are dummy variables. Reference variable is low strain.

Each model is presented without intercepts and adjusted for age, smoking, education and occupational status.

Estimates are given for the regression coefficient, confidence interval (95%) and p-value.

Table 4 illustrates the multiple linear regressions between the ERI-ratio and CHD risk factors. Men had increased scores for triglycerides (p = 0.04), and BMI (p = 0.01), while women had higher HDL-cholesterol (p = 0.02). Table 5 shows the multiple linear regression analysis between the complementary ERI model and CHD risk factors. There was a link between high effort-low reward (ERI-1) and increased BMI for men. Notably, this complementary method also showed that the female group reporting high effort-high reward jobs had beneficial lower values for triglycerides (p = 0.03) and increased scores for HDL-cholesterol (p = 0.02). Additionally women with low effort-low reward jobs had decreased SBP. 

**Table 4:** Multiple linear regression for ERI ratio and CHD risk factors

	**DBP**	**SBP**	**Triglycerides**	**Total cholesterol**	**HDL-cholesterol**	**LDL-cholesterol**	**BMI**
**Men**							
Estimate	0.3	0.7	0.2	0.1	−0.02	−0.2	1.2
CI (95%)	(−1.9 ; 2.5)	(−2.7 ; 4.1)	(0.01 ; 0.3)	(−0.4 ; 0.1)	(−0.1 ; 0.1)	(−0.4 ; 0.02)	(0.5 ; 1.9)
p-value	0.77	0.68	**0**.**04**	0.22	0.65	0.08	**0**.**01**
**Women**							
Estimate	0.7	−2.0	0.04	−0.1	−0.1	−0.02	0.5
CI (95%)	(−1.4 ; 2.7)	(−5.4 ; 1.4)	(−0.1 ; 0.1)	(−0.3 ; 0.1)	(−0.2 ; -0.1)	(−0.2 ; 0.2)	(−0.2 ; 1.3)
p-value	0.53	0.25	0.48	0.25	**0**.**02**	0.78	0.17

Quartiles were created from the distribution of ratio scores between effort and reward. The highest quartile of the distribution was compared to the lowest quartile.

Each model is presented without intercepts and adjusted for age, smoking, education and occupational status.

Estimate is given for the regression coefficient, confidence interval (95%) and p-value.

**Table 5:** Multiple linear regression for the complementary ERI model and CHD risk factors

	** ERI-1(High effort-low reward)**		** ERI-2(High effort-high reward)**		** ERI-3(Low effort-low reward)**	
	**Estimate(95%CI)**	**P-value**	**Estimate(95%CI)**	**P-value**	**Estimate(95%CI)**	**P**-**value**
DBP (mmHg)						
Men	−1.0 (−3.6 ; 1.5)	0.43	−1.2 (−3.7 ; 1.3)	0.35	−0.7 (−3.5 ; 2.2)	0.64
Women	−1.6 (−4.0 ; 0.8)	0.19	−1.7 (−4.8 ; 1.4)	0.29	−2.2 (−4.8 ; 0.4)	0.10
SBP (mmHg)						
Men	−0.7 (−4.6 ; 3.2)	0.73	−1.5 (−5.4 ; 2.3)	0.44	−2.0 (−6.3 ; 2.3)	0.36
Women	−3.8 (−7.7 ; 0.1)	0.06	−4.2 (−9.3; 0.9)	0.11	−4.5 (−8.8 ; -0.2)	**0**.**04**
Triglycerides						
Men	0.1 (−0.1 ; 0.3)	0.23	0.1 (−0.06 ; 0.3)	0.21	−0.02 (−0.2 ; 0.2)	0.86
Women	0.01 (−0.1 ; 0.1)	0.82	−0.2 (−0.3 ; - 0.02)	**0**.**03**	−0.08 (−0.2 ; 0.06)	0.24
Total cholesterol (mmol/L)						
Men	−0.01 (−0.3 ; 0.2)	0.91	−0.1 (−0.4 ; 0.1)	0.33	0.04 (−0.2 ; 0.3)	0.78
Women	0.04 (−0.3 ; 0.2)	0.83	−0.01 ( −0.3 ; 0.3)	0.97	−0.01 (−0.2 ; 0.3)	0.78
HDL-cholesterol (mmol/L)						
Men	−0.002 (−0.1 ; 0.09)	0.96	−0.06 (−0.2 ; 0.04)	0.22	0.03 (−0.1 ; 0.1)	0.60
Women	−0.02 (−0.1 ; 0.1)	0.76	0.2 (0.03 ; 0.3)	**0**.**02**	0.02 (−0.1 ; 0.1)	0.75
LDL-cholesterol (mmol/L)						
Men	−0.05 (−0.3; 0.2)	0.69	−0.1 (−0.3 ; 0.1)	0.35	0.1 (−0.2 ; 0.3)	0.85
Women	−0.01 (−0.2 ; 0.2)	0.89	−0.1 (−0.4 ; 0.2)	0.50	0.1 (−0.2 ; 0.3)	0.61
BMI (kg/m2)						
Men	0.9 (0.1 ; 1.7)	**0**.**03**	0.5 (−0.3 ; 1.3)	0.22	0.5 (−0.8 ; 1.1)	0.76
Women	0.7 (−0.2 ; 1.5)	0.14	−0.6 (−1.7 ; 0.5)	0.29	0.5 (−0.4 ; 1.5)	0.26

Each model is presented without intercepts and adjusted for age, smoking, education and occupational status.

ERI-1 (High Effort-Low Reward), ERI-2 (High Effort-High Reward) and ERI-3 (Low Effort-Low Reward) are dummy variables.

Reference variable is ERI-4 (Low effort-High Reward).

Estimates are given for the regression coefficient, confidence interval (95%) and p-value.

## Discussion

In accordance with previous studies, links between psychosocial work environment and CHD risk factors were inconsistent. Somewhat surprisingly there were health beneficial scores for women reporting high effort-high reward. These subjects had lower triglycerides and elevated HDL-cholesterol compared to those exposed to low effort-high reward.

Otherwise, results when analysing JDC concurred with earlier research. For men, JDC variables were related to elevated scores in DBP, SBP, triglycerides, total cholesterol and LDL-cholesterol, while for women all relationships tested null. Regarding relationships between ERI variables and CHD risk factors most analysis tested null. The ERI analysis using the ERI-ratio showed that men reporting effort-reward imbalance had higher BMI and triglycerides, while women had lowered HDL-cholesterol.

The gender differences in the association between JDC variables and ill-health measure supports earlier research which has shown strong links between high strain and impaired health for men [2,4-8], but weak or no relationships for women [14,17,32]. Unlike earlier findings, where active jobs were deemed as more hazardous for female health than high strain [16,17,33] this study found no links between CHD risk factors and active job exposure for women. Despite concordance with earlier findings, this still raises questions of gender differences for associations between psychosocial work exposure and health.

The JDC model was originally developed when investigating typical male blue-collar working conditions, [1,4] and is therefore sometimes considered to best assess task-oriented work qualities, while emotional demands such as caring for patients, are perhaps not equally well captured. Given that in this sample, men were more frequently employed in blue-collar jobs than women, their work conditions might be more accurately evaluated by JDC items. Accordingly, high strain occupations dominated by women, like health care, may not be properly reflected using this structure. Differences between participant’s occupational categorization illustrated in Table 6. 

**Table 6:** Descriptive statistics of job categories for men and women according to ISCO-88

**Job category**	**Men**	**Women**
	**n(%)**	**n(%)**
White-collar work		
Executive work	78 (12.2)	39 (5.8)
High skilled academics	160 (25.1)	166 (24.9)
Low skilled academics	101 (17.8)	118 (17.7)
Office and client service work	49 (7.7)	130 (19.5)
Care and retail service work	26 (4.1)	134 (20.1)
**Total**	**372** (**68**.**7**)	**507** (**90**.**2**)
Blue-collar work		
Farming, gardening and foresting	6 (0.9)	2 (0.3)
Construction and installation work	97 (15.2)	6 (0.9)
Machine operators and transport	60 (9.4)	8 (1.2)
Work that does require any training	35 (5.5)	39 (5.8)
**Total**	**170** (**31**.**3**)	**55** (**9**.**8**)

Evidence of gender dissimilarities could also be found when exploring JDC exposure combined with occupational status. In a study by Tsusumi and colleagues [34] men with high strain and low status jobs had an elevated risk for stroke. Women holding managerial jobs combined with high strain exposure had a substantially higher risk of developing stroke than other women. Findings like this could further emphasize that the JDC model may be most relevant for assessing work environment for men in blue-collar jobs. These results could also suggest that work aspects captured by JDC variables are not the key components regarding women’s health. Instead, social dimensions might be more important. Since the JDC has a more task oriented approach and the ERI model involves social aspects, this could perhaps explain the less distinct gender differences in the ERI analysis. Yet another aspect involves how the reward variable contains aspects of job security and organizational injustice [27]. Although gender equality has improved in recent decades there are likely still differences in how both are treated in the work place. This is perhaps most pronounced in job rewards such as salary and job promotions. Sensitivity to occupational justice could also contribute to why women are more linked to ERI measures than to JDC.

To some extent, the results for women also differed regarding the ERI analysis when the complementary model was used. Although results for the alternative model resembled the outcomes for the ratio analysis, health beneficial results for women exposed to high effort-high rewards (ERI-2) were also observed. In addition to the high effort-high reward qualities, this group also contained the highest proportion of university educated subjects and white-collar workers. Given these characteristics, the women in this group could possibly represent subjects with a strong career focus, who seemingly receive appropriate reward for their labours which then serve to benefit health. But it is also possible that this categorization of subjects also creates groups in terms of job status. According to Marmot [35] different jobs connect to different lifestyle values such as diet and exercise. It might be that within these high status jobs there are social norms that encourage health-enhancing ideals that alongside the positive work environment contribute to increased health measures. On the other hand if this was the sole explanation to health beneficial relationships in the ERI-2 group, the results among men should also illustrate these differences. Taking these assumptions into consideration, a suggestion for future studies using the JDC and ERI structures could be to stratify for job status.

A major limitation in this study, apart from the cross-sectional design, which in itself constituted a major limitation, was the constitution of the sample. The study was comprised of randomly-selected subjects from greater Gothenburg and surrounding areas, as has previously been reported in a selection bias study made by Strandhagen and colleagues [36]. This study illustrated that those declining participation tended to be men, younger, have lower education and to originate from outside Scandinavia. The study also illustrates that those with lower education tend to have a worse risk factor pattern with respect to triglycerides, HDL-cholesterol, LDL-cholesterol, hypertension and BMI. Given this information, added to the fact that young and middle aged men tend to have more heart disease; it is possible that the sample is biased towards a healthier sample.

This study was also limited to those currently working, which could have contributed to a healthy-worker survivor [37]. This refers to the tendency that a sample might be biased since sick or injured subjects are not able to work. Due to the study design, all subjects on sick leave or early retirement were excluded from analyses. It is possible that some subjects were not working due to work related ill-health. Since not providing information on their work environment, the sample might be skewed towards more healthy participants and better psychosocial work scores. It is difficult to know to what level this holds true, but if many subjects with work-related sickness were not included, it undermines the study’s internal validity. It could possibly also conceal relationships between psychosocial variables and biomarkers. In future studies it could be useful to measure the latest work conditions for early retired or those on longer sick leave. The study design also relied on the use of sum scores and consequently we excluded all subjects with missing values in JDC or ERI items. However, analyses comparing mean for the demand, control, effort and reward variables between those included in the study and those that were excluded showed very minor differences.

Another weakness in this study is the lack of measures of social support at work. Arguing for a lack of a social dimension in the JDC model [38] the structure was complemented with the social support factor, which is thought to buffer psychological distress. Findings from the Whitehall II studies [39] show that women tend to have a wider range of close social relations among both colleagues and friends, and greater satisfaction with those relationships. Given women’s stronger association between social dimensions and health, as well as the buffering effect of social support, this variable might serve as a mediator. Including of such a variable could have been a complement in understanding the gender differences in the result.

Yet another methodological consideration involves the ERI analysis. The effort-reward imbalance at work questionnaire is designed for samples with similar work. As a consequence, the item “My work is physically demanding” is to be either included or removed depending on a predominantly white- or blue-collar group of subjects. This present sample does, however, include a broad variety of occupations. In a study by Siegrist and colleagues [40] it is argued that the item measuring physical work load should be removed for samples consisting of mostly white-collar workers. Since the sample in the present study comprised a majority of white-collar workers and considering the choice of method by the developer of the ERI model it was decided to carry out the analysis.

Despite these limitations our findings indicate that men and women may differ with respect to relationships between psychosocial work exposure and health.

## Conclusions

Although most analyses tested null, this descriptive study illustrated some differences between men and women regarding the associations between psychosocial exposure at work and CHD risk factors. Possible explanations for these gender differences are that men and women are stressed by different psychosocial variables. It could also be an indicator that the JDC and ERI correspond differently to typically male and female work environments. One aim of cross-sectional studies is to assess associations aiming at general hypotheses, which can be addressed further in other types of studies. Given these indications of different relationships between psychosocial features and CHD risk factors, future longitudinal studies should investigate these relationships relationship, stratifying for gender and job status.

## Competing interests

The author(s) declare that they have no competing interests.

## Authors’ contributions

MS - Interpretation of relationship between psychosocial variables and cardiovascular heart disease risk factors data, statistical analysis, drafting the manuscript, final approval of the manuscript to be published. JH – Carried out the main design of the statistical analysis, critically revising the manuscript, final approval of the manuscript to be published. LL - Principal investigator of the InterGene main study and the psychosocial module, critically revising the manuscript, final approval of the manuscript to be published. AR - Principal investigator of the InterGene study, carried out main interpretations of cardiovascular heart disease risk factors data, critically revising the manuscript, final approval of the manuscript to be published. KT - Principal investigator of the Adonix study, statistical analysis, interpretation of data, critically revising the manuscript, final approval of the manuscript to be published. All authors read and approved the final manuscript.

## Pre-publication history

The pre-publication history for this paper can be accessed here:

http://www.biomedcentral.com/1471-2458/12/1102/prepub
